# Neonatal Androgenization Exacerbates Alcohol-Induced Liver Injury in Adult Rats, an Effect Abrogated by Estrogen

**DOI:** 10.1371/journal.pone.0029463

**Published:** 2011-12-20

**Authors:** Whitney M. Ellefson, Ashley M. Lakner, Alicia Hamilton, Iain H. McKillop, Herbert L. Bonkovsky, Nury M. Steuerwald, Yvette M. Huet, Laura W. Schrum

**Affiliations:** 1 Department of Biology, University of North Carolina at Charlotte, Charlotte, North Carolina, United States of America; 2 Molecular Biology Core Facilities, Carolinas Medical Center, Charlotte, North Carolina, United States of America; 3 Department of General Surgery, Carolinas Medical Center, Charlotte, North Carolina, United States of America; 4 Department of Internal Medicine, Carolinas Medical Center, Charlotte, North Carolina, United States of America; 5 Liver-Biliary-Pancreatic Center, Carolinas Medical Center, Charlotte, North Carolina, United States of America; Clermont Université, France

## Abstract

Alcoholic liver disease (ALD) affects millions of people worldwide and is a major cause of morbidity and mortality. However, fewer than 10% of heavy drinkers progress to later stages of injury, suggesting other factors in ALD development, including environmental exposures and genetics. Females display greater susceptibility to the early damaging effects of ethanol. Estrogen (E2) and ethanol metabolizing enzymes (cytochrome P450, CYP450) are implicated in sex differences of ALD. Sex steroid hormones are developmentally regulated by the hypothalamic-pituitary-gonadal (HPG) axis, which controls sex-specific cycling of gonadal steroid production and expression of hepatic enzymes. The aim of this study was to determine if early postnatal inhibition of adult cyclic E2 alters ethanol metabolizing enzyme expression contributing to the development of ALD in adulthood. An androgenized rat model was used to inhibit cyclic E2 production. Control females (Ctrl), androgenized females (Andro) and Andro females with E2 implants were administered either an ethanol or isocalorically-matched control Lieber-DeCarli diet for four weeks and liver injury and CYP450 expression assessed. Androgenization exacerbated the deleterious effects of ethanol demonstrated by increased steatosis, lipid peroxidation, profibrotic gene expression and decreased antioxidant defenses compared to Ctrl. Additionally, CYP2E1 expression was down-regulated in Andro animals on both diets. No change was observed in CYP1A2 protein expression. Further, continuous exogenous administration of E2 to Andro in adulthood attenuated these effects, suggesting that E2 has protective effects in the androgenized animal. Therefore, early postnatal inhibition of cyclic E2 modulates development and progression of ALD in adulthood.

## Introduction

Alcoholic liver disease (ALD) affects millions of people worldwide and is a major cause of morbidity and mortality [1]. ALD encompasses varying degrees of hepatic injury progressing from steatosis (fatty liver) to more advanced damage, including hepatic inflammation and cell death, fibrosis/cirrhosis and hepatocellular carcinoma [2]. However, less than 10% of heavy drinkers progress to later stages of injury, suggesting other contributing factors in development of severe liver injury due to excessive alcohol consumption. Of these factors, health status (obesity), environmental exposures (smoking, diet, endocrine disruptors) and genetics (sex differences) influence development and progression of ALD [3].

Sex differences are observed in rodent models of ALD, with females displaying greater susceptibility to the detriments of alcohol than males [4], [5], [6]. Since decreasing estrogen (E2) levels [*via* antiestrogens or ovariectomy (OVX)] [7], [8] protects females from ethanol-induced liver injury, E2 is implicated in sex differences observed in ALD. However, in these reports, E2 manipulation was initiated post-pubertally (antiestrogens) or at 4 weeks of age (OVX). No studies to date have determined the contribution of cyclic E2 in the development of ALD. Additionally, OVX studies cannot exclude the possibility that other ovarian factors besides E2 (e.g. progesterone, inhibin) may play a role in development and progression of ALD. Gonadal sex steroid hormone levels are regulated by the hypothalamic pituitary gonadal (HPG) axis, which is disrupted by neonatal androgenization. Further, gonadal hormone production, regulated by the HPG axis, programs sex-specific expression of hepatic enzymes during pubertal development and can profoundly affect adult liver physiology [9]. Perinatal hormonal imprinting of hepatic enzymes including members of the cytochrome P450 family and those involved in steroid metabolism has been shown [9], [10].

Alterations in expression of ethanol metabolizing enzymes have been implicated in predisposition to ALD [2]. Following acute and chronic ethanol consumption, hepatocytes (liver parenchyma) are the principal site of ethanol metabolism. Classically, ethanol metabolism occurs *via* alcohol dehydrogenase (ADH), the microsomal ethanol oxidizing system, comprised predominantly of inducible cytochrome P450 2E1 (CYP2E1) and catalase [11]. Metabolism by ADH and CYP2E1 generates reactive oxygen species, promoting lipid peroxidation, protein adduct formation and collagen synthesis, the major mechanisms of damage in ALD progression [1]. The liver counteracts the deleterious effects of oxidative stress *via* increased antioxidant defense mechanisms (including superoxide dismutase, catalase and glutathione peroxidase), providing protection against ALD development [12]. During chronic ethanol exposure, the balance between pro-oxidants and anti-oxidants may favor pro-oxidants, thus rendering the cell susceptible to oxidative stress [1]. Despite increased oxidative stress by ethanol-induced CYP2E1, previous studies report CYP2E1 expression may not be the predominant mechanism of alcohol-induced damage, suggesting possible involvement of other CYP450 family members in ethanol metabolism and ALD progression [13]. CYP1A2 is also capable of metabolizing ethanol, and interestingly, is induced in a sex-specific manner with potential regulation by E2 [14], [15], [16], [17]. Therefore, sex-specific ethanol metabolizing enzymes may contribute to sexual dimorphism in ALD.

In the present study, neonatal androgenization was used as a model to examine the contribution of adult cyclic E2 in the development and progression of ALD. Neonatal androgenized animals undergo persistent oestrus with non-ovulating polyfollicular ovaries and, thus, leads to constant levels of E2 secretion in the adult (no E2 cyclicity) [18]. The androgenization model was selected over OVX in order to minimize effects of prepubertal fluctuations of E2 while still providing a constant basal amount of E2, similar to levels observed on the morning of proestrus. Since androgenization programs “male-like” specific gonadotropin patterns and blocks E2 cyclicity, we anticipated alcohol-induced liver damage to be less severe than in normal females and to be more closely aligned with that of normal males. Additionally, because E2 is implicated in sex differences observed in ALD, androgenized animals administered constant exogenous E2 (via E2-packed silastic implants) were expected to display increased liver injury. Contrary to what we expected, our study demonstrated that neonatal androgenization exacerbated alcohol-induced liver injury mediated, in part, by increased oxidative stress and profibrotic gene expression and that constant E2 administration during adulthood was able to abrogate this effect. Additionally, CYP2E1 protein expression was decreased by androgenization suggesting that alcohol metabolism through this enzyme does not contribute to the damaging effects. Therefore, these studies provide further insight into the contribution of cyclic E2 and adult exposure to continuous E2 in alcohol-induced liver injury, and an improved understanding of sexual dimorphism in ALD.

## Materials and Methods

### Materials

Lieber-DeCarli liquid diets were purchased from Dyets, Inc (Bethlehem, PA). Silastic tubing (I.D.×O.D.: 0.040″×0.085″) was purchased from Dow Corning (Midland, MI). Testosterone and estrogen (estradiol-17β) were purchased from Sigma Chemical Co. (St. Louis, MO). A BCA kit to measure protein concentrations was purchased from Pierce Biotechnology, Inc (Santa Cruz, CA) and 540 nm absorbance was measured on a BioTek Synergy HT Multi-Detection Microplate Reader (BioTek Instruments, Winooski, VT) using Gen5 Analysis Software. For Western blot analyses, antibodies against cytochrome P450 2E1 (CYP2E1), cytochrome P450 1A2 (CYP1A2) and glyceraldehyde-3-phosphate dehydrogenase (GAPDH) were purchased from Millipore (Billerica, MA). Antibody against 4-Hydroxynonenal (4-HNE) was purchased from Alpha Diagnostic Intl (San Antonio, TX).

### Animals and Experimental Procedures

**Figure 1:**
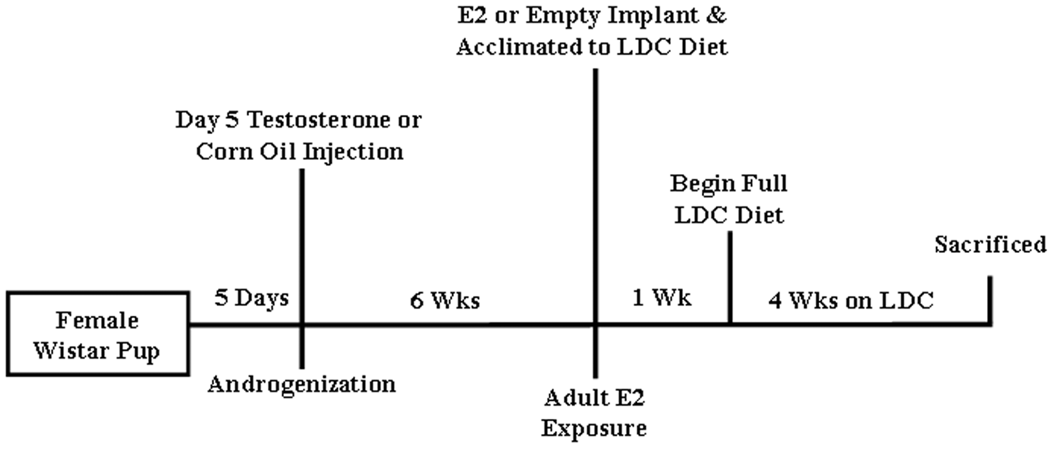
Summary of experimental design. Day 5 old Wistar female pups were administered a single subcutaneous injection of testosterone (1.0 mg) in 0.02 ml corn oil (androgenized) or corn oil (control). After 6 weeks, androgenized and control females were subcutaneously implanted with empty silastic tubing or tubing packed with 17β-estradiol. One day after receiving implants rats were acclimated to the ethanol (E) or control (C) Lieber-DeCarli (LDC) diet. Animals were administered the full E-LDC (36% calories from ethanol) or C-LDC (isocalorically-matched) for 4 weeks and then sacrificed. Five animals were used for each group.

Female Wistar rats (Charles River Laboratories, Wilmington, MA) were used in this study. For inhibition of cyclic E2, an androgenized rat model was used. Day 5 female pups (birth  =  day 1) were administered a single subcutaneous injection of 1.0 mg of testosterone in 0.02 ml corn oil (androgenized) or a single injection of corn oil (control) (Figure 1), permanently inhibiting cyclic E2 production, but maintaining basal levels of E2. Considering that liver pathology will be assessed due to chronic effects of androgenization and alcohol, control animals were not staged according to cycle at time of harvest. At seven weeks (approximately 170–220g), androgenized female rats were exposed to constant elevated estrogen (E2) levels *via* silastic implants (packed with 17β-estradiol powder) or empty silastic implants placed subcutaneously on back of the neck. One day after receiving implants, rats were assigned to experimental groups: control or ethanol-containing Lieber-DeCarli (LDC) liquid diet (Figure 1). In the ethanol-LDC group (E-LDC), the amount of ethanol was sequentially increased during the first week of feeding until 36% of total dietary calories from ethanol were achieved [19]. The control-LDC (C-LDC) group received an isocaloric liquid diet in which carbohydrates were substituted for ethanol. One week post ethanol acclimatization, rats were administered full ethanol or control diet for four weeks. Water was provided *ad libitum*. All experimental procedures were approved by the Institutional Animal Care and Use Committee of University of North Carolina at Charlotte (IACUC ID: 09-009.0) and performed in accordance with the National Institutes of Health guidelines for Care and Use of Laboratory Animals. After four weeks of ethanol feeding, animals were sacrificed and tissues were harvested. Blood was collected and plasma obtained by centrifugation (1,500×g, 15 minutes, 4°C) and stored at −80°C. Liver tissue was either snap-frozen in liquid nitrogen and stored at −80°C for protein and RNA analyses or fixed in neutral-buffered formalin for histology.

### Plasma Estradiol

Plasma estradiol levels were measured using a high-sensitivity radioimmunoassay (Beckman Coulter, Brea, CA) with a sensitivity of 2 pg/ml. Estradiol assays were run in duplicate for each individual plasma sample, taken at sacrifice. The assay has an intra-assay coefficient of variation of 8.9%.

### Liver Histology

Liver tissue was harvested and fixed in 10% neutral buffered formalin for histological analysis. Sections (4 µm) from formalin-fixed paraffin-embedded tissues were cut and deparaffinized with three changes of xylene (10 minutes), cleared with two changes of 100% ethanol and rehydrated with two changes of 95 and 70% ethanol and water. For Hematoxylin and Eosin (H&E) staining, slides were stained in Mayer's Hematoxylin for 15 minutes and rinsed for 15 minutes in water, followed by an 80% ethanol rinse and incubated in Eosin Y for 15 seconds. For 4-HNE staining, slides were incubated with 4-HNE antibody at 1∶500 dilution for 1 hour at room temperature and secondary antibody for 30 minutes, then developed with DAB substrate (Vector Laboratories, Burlingame, CA). Tissues were dehydrated and cleared through 95% and 100% ethanol and xylene. Coverslips were then applied with Permount.

### Triglyceride Measurement

Liver tissue triglyceride concentrations were measured using a Triglyceride Quantification Kit (Biovision, Mountain View, CA). Fifty mg of liver was homogenized in a 5% NP-40 buffer, and assay was performed according to manufacturer's directions. Absorbance was measured on a BioTek Synergy HT Multi-Detection Microplate Reader using Gen5 Analysis Software.

### Thiobarbituric Acid Reactive Substances Assay

Thiobarbituric Acid Reactive Substances (TBARS) in tissue homogenates were quantified using a TBARS Assay Kit through determination of malondialdehyde (MDA) formation (Cayman Chemical Company, Ann Arbor, MI). Tissue was homogenized in RIPA buffer [1% (v/v NP-40, 0.5% (w/v) sodium deoxycholate, 0.1% (w/v) SDS, 0.5 mM phenylmethylsulfonyl fluoride (PMSF), 0.05 mM Na_3_VO_4_, 2 µg/ml aprotinin in phosphate-buffered saline (PBS)], sonicated, centrifuged at 16,000×g for 10 minutes at 4°C and supernatants stored at −80°C. Assay was performed according to manufacturer's directions. Protein concentrations were measured using a BCA kit and TBARS were normalized to total protein.

### Superoxide Dismutase Activity Assay

Superoxide dismutase (SOD) activity was measured in tissue homogenates using an SOD Assay Kit (Cayman Chemical Company). Tissue was homogenized in HEPES buffer (1 mM EGTA, 210 mM mannitol and 70 mM sucrose, pH 7.2), centrifuged at 1,500×g for 5 minutes at 4°C and supernatant stored at −80°C. Assay was performed according to manufacturer's directions. Protein concentrations were measured using a BCA kit and SOD activity normalized to protein.

### Catalase Activity Assay

Catalase activity was measured in tissue homogenates using a Catalase Assay Kit (Cayman Chemical Company). Tissue was homogenized in potassium phosphate buffer (50 mM potassium phosphate, 1 mM EDTA, pH 7.0), then centrifuged at 1,500×g for 5 minutes at 4°C and supernatant stored at −80°C. Assays were performed according to manufacturer's directions on a 96-well plate. Protein concentrations were measured using a BCA kit and catalase activity normalized to total protein.

### Western Blotting Analyses

Total tissue lysates were prepared in lysis buffer (50 mM Tris-HCL, 0.1 mM EDTA, 0.2% (w/v) sodium dodecyl sulfate, and protease inhibitor cocktail), sonicated, and centrifuged (16,000×g, 10 minutes, 4°C) and supernatant stored at −80°C as previously described [19]. Protein concentration was measured using a BCA method and protein samples boiled for 5 minutes with an equal amount of NuPAGE® denaturing buffer (Invitrogen, Carslbad, CA). Protein samples were resolved on a 10% (w/v*)* polyacrylamide gel prior to transfer onto nitrocellulose membrane. Ponceau S (Sigma Aldrich, St. Louis, MO) staining was used to determine equal protein loading and to assess transfer quality. Membranes were blocked in 5% non-fat dry milk in TBS-T (25 mM Tris-HCl, pH 8.0, 144 mM NaCl, 0.1% Tween 20), washed with TBS-T and incubated overnight at 4°C with 1∶1,000 dilution of primary antibody and 1∶5,000 dilution of HRP-conjugated secondary antibody. Membranes were washed and bound enzymes detected with enhanced chemiluminescence (ECL) solution (Pierce Biotechnology, Inc). Densitometric quantification of band intensity was performed using a densitometric analysis program (Quantity One, Bio-Rad Laboratories, Inc; Hercules, CA). Glyceraldehyde-3-phosphate dehydrogenase (GAPDH) was used as a control.

### RNA Isolation and Real-Time Polymerase Chain Reaction (qRT-PCR) Analyses

Total RNA was isolated from liver tissue using TRIzol reagent (Gibco-BRL, Gaithersburg, MD). For collagen 3α1 (*col3α1*), interleukin-6 (*IL-6*) and *cyp2e1* mRNA quantitation, first-strand complementary DNA was synthesized using an iScript™ cDNA synthesis kit (Bio-Rad, Inc), and 50 ng of final product was used as template for PCR. qRT-PCR was performed using TaqMan® Probe-Based Detection (Life Technologies, Carlsbad, CA) with an ABI Prism 7500 Fast Real-Time PCR System using Taqman® Gene Expression Master Mix (Life Technologies) as previously reported [20]. For *cyp1a2* mRNA expression, total RNA was reverse-transcribed using Superscript II reverse transcriptase (Promega, Madison, WI), and qRT-PCR was conducted using IQ SYBR Green Supermix (Bio-Rad, Inc) as described previously [21]. Fold change values were calculated by comparative cycle threshold (Ct) value analysis after normalizing to GAPDH mRNA.

### Statistical Analyses

All data are presented as mean ± SEM. In the animal studies, for each treatment group, five animals were used. Data were analyzed using two-way ANOVAs testing both sex hormone status and diet followed by Kruskal-Wallis post-hoc test for pairwise comparisons. *A priori* a, P<0.05 was considered significant. Standard square root transformation was performed prior to statistical analysis for Cyp2E1 protein expression for E-LDC group.

## Results

### Androgenization Exacerbates Ethanol-Induced Hepatic Steatosis

**Figure 2:**
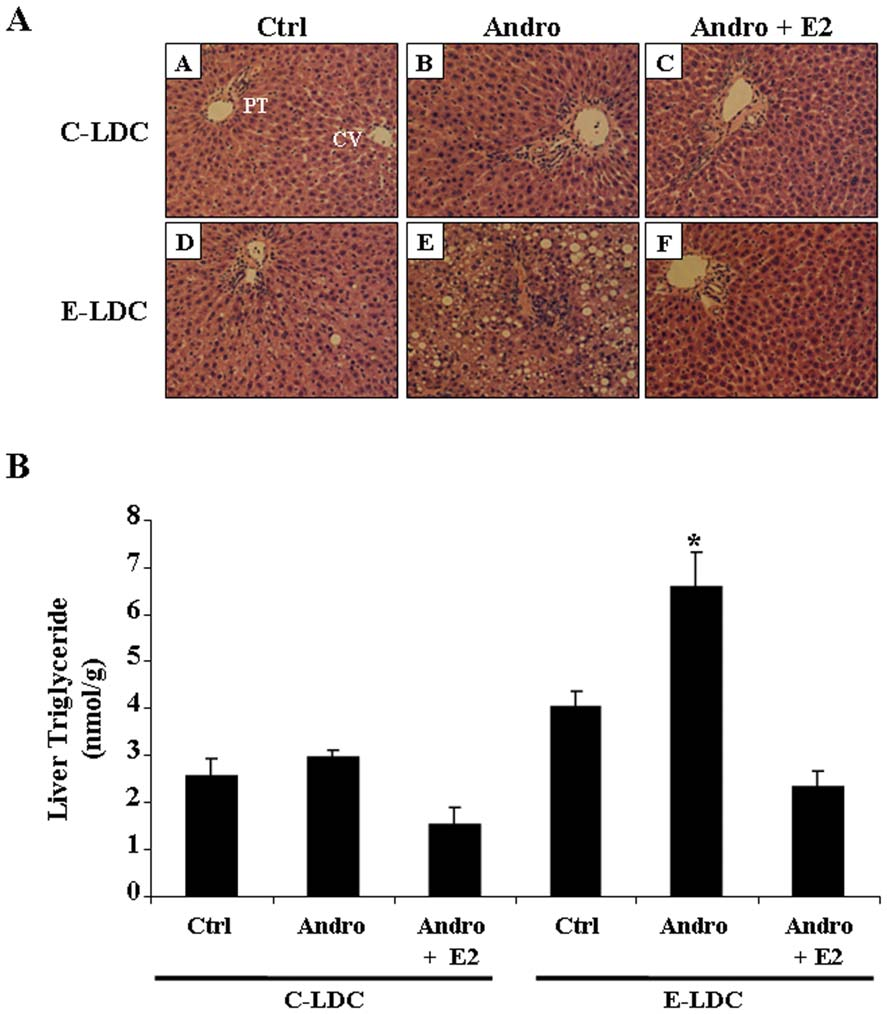
Effects of ethanol and androgenization on liver damage. A. Representative images of H&E stained formalin-fixed paraffin-embedded liver tissue from rats maintained on control Lieber-DeCarli (C-LDC, top panels) or ethanol-LDC (E-LDC, bottom panels) diet. Panels A and D, control females (Ctrl); B and E, androgenized females (Andro); panels C and F, Andro + estrogen (Andro + E2). PT, portal triad; CV, central vein. B. Hepatic tissue triglyceride concentrations. Liver tissue (50 mg) was homogenized in 5% NP-40 buffer and triglycerides were measured for all groups. Data are presented as mean values ± SEM. *P<0.05 compared to all groups.

Representative H&E stained tissue sections from each experimental group are shown in Figure 2A. No significant histological differences were observed between the three groups maintained on C-LDC diets (Fig. 2A, panels A–C). However, increased micro- and macrovesicular steatosis was observed in control female (Ctrl) livers of rats maintained on E-LDC (panel D) as compared to pair-matched animals maintained on C-LDC diet (panel A). Andro rats on the E-LDC diet (panel E) had increased steatosis compared to the Ctrl on E-LDC (panel D). Andro animals exposed to constant E2 exhibited improved liver histology with a marked reduction in steatosis (panel F). To further validate steatosis observed by H&E staining, liver tissue triglycerides were quantified. These data demonstrate androgenization increased tissue triglyceride concentrations compared to Ctrl on E-LDC diet and exogenous E2 abrogated this effect (Fig. 2B). No significant differences in triglycerides were observed in animals on C-LDC. Elevated levels of E2 were successfully released from implants as indicated by plasma estradiol concentrations (Table 1). Additionally, as previously demonstrated [22], [23], [24], [25] basal levels of estradiol were also observed in the androgenized animals (Table 1).

**Table 1:** Plasma Estradiol Concentrations.

ExperimentalGroup	PlasmaEstradiol (pg/ml)
Ctrl	36.11±4.8
Andro	30.78±5.8
Andro + E2	81.17±12.1
Ctrl + EtOH	23.42±2.6
Andro + EtOH	25.72±6.6
Andro + E2 + EtOH	571.16±52.3

Plasma 17β-Estradiol was measured by a radioimmunoassay for Ctrl, Andro, Andro + E2 rats maintained on C-LDC or E-LDC diets. Data are presented as mean values ± SEM.

### Androgenization Exacerbates Ethanol-Induced Oxidative Stress

**Figure 3:**
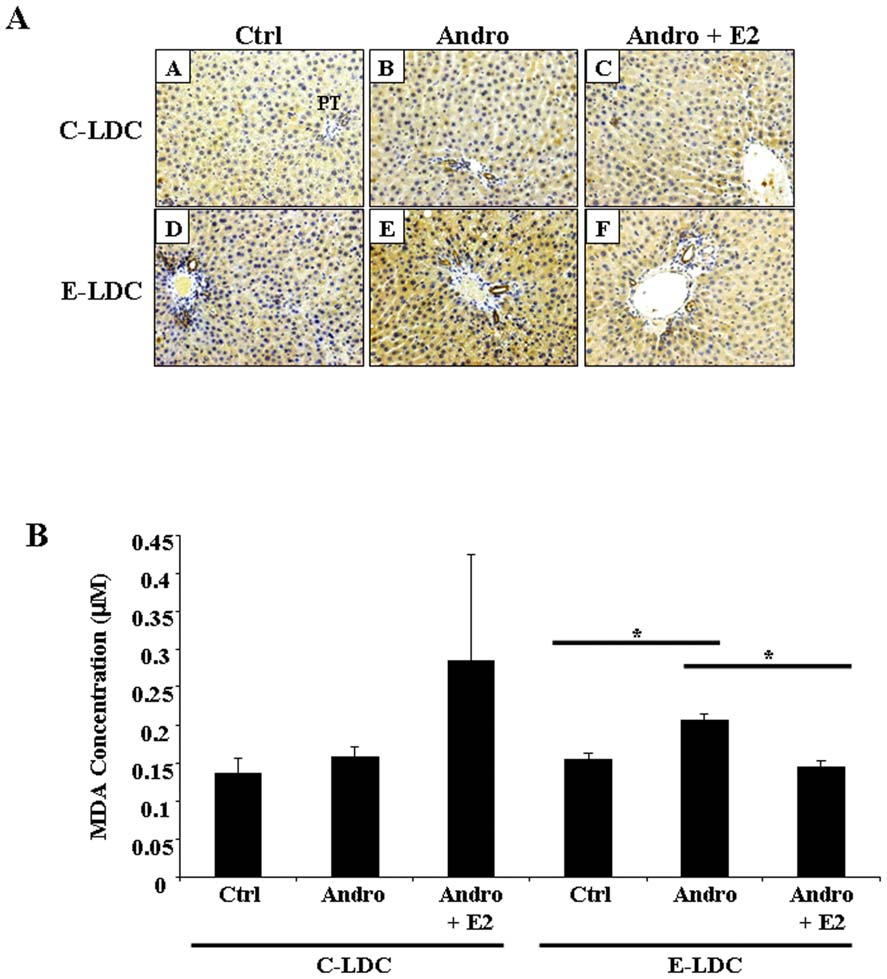
Effects of ethanol and androgenization on oxidative stress. A. Representative images of 4-HNE stained formalin-fixed paraffin-embedded liver tissue from rats maintained on control Lieber-DeCarli (C-LDC, top panels) or ethanol-LDC (E-LDC, bottom panels) diet. Panels A and D, control females (Ctrl); B and E, androgenized females (Andro); panels C and F, Andro + estrogen (Andro + E2). PT; portal triad. Caramel brown staining indicating 4-HNE positive cells. B. Hepatic lipid peroxidation was assessed by Thiobarbituric Acid Reactive Substances (TBARS) assay. Liver tissue was homogenized and malondialdehyde (MDA) formation measured. TBARS were normalized to total protein. Data are presented as mean values ± SEM. *P<0.05.

Oxidative stress was assessed by 4-HNE staining (Fig. 3A) and TBARS (Fig. 3B) assay. Androgenized animals on E-LDC showed increased 4-HNE positive cells (Fig. 3A, panel E) as indicated by caramel brown cytoplasmic staining, particularly associated with areas of increased steatosis, compared to Ctrl on E-LDC (Fig. 3A, panel D). Exogenous E2 decreased staining comparable to Ctrl on E-LDC (Fig. 3A, panel F). No difference in 4-HNE staining was observed among the three groups on the C-LDC diet (Fig. 3A, panels A–C). As a quantitative marker of oxidative stress, MDA concentrations were increased in Andro compared to Ctrl on E-LDC, and exogenous E2 abrogated this effect. No significant differences in TBARS were observed in animals on C-LDC.

### Superoxide Dismutase Activity is Reduced in Androgenized and Ethanol-Fed Rats

**Figure 4:**
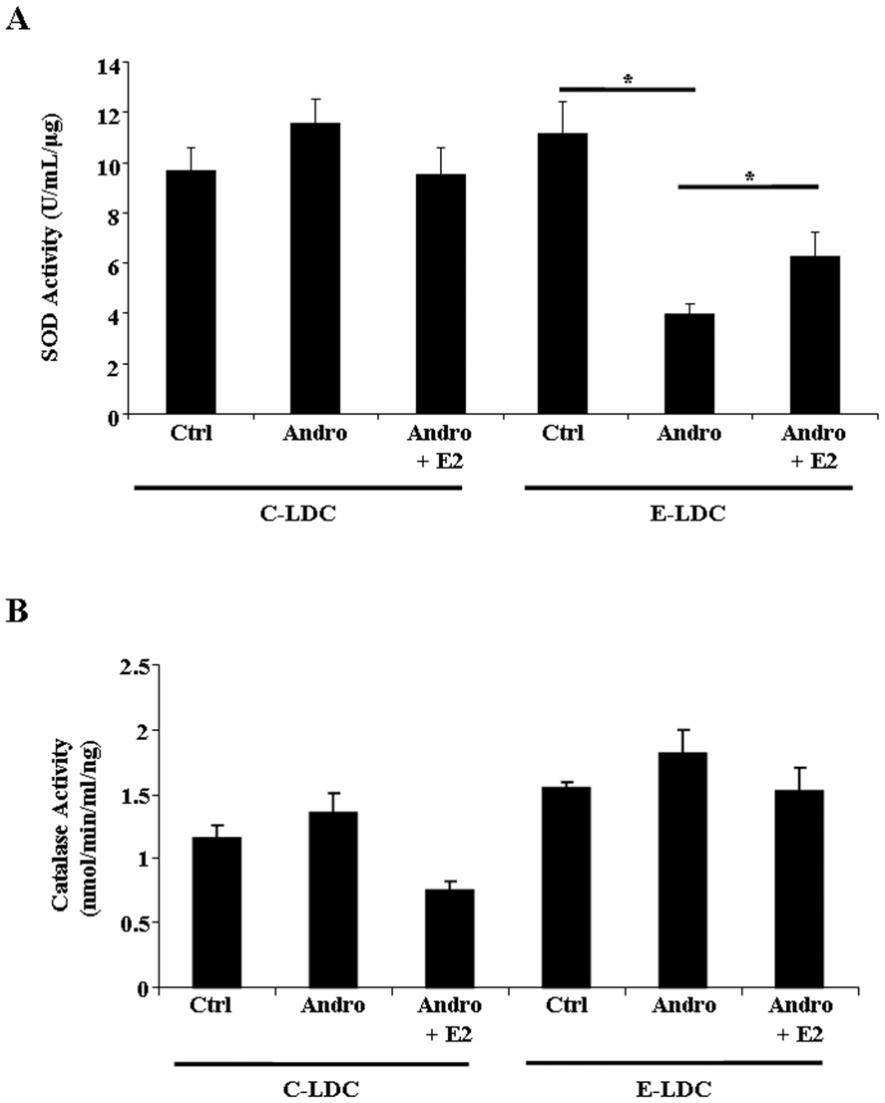
Effects of ethanol and androgenization on antioxidant defense activity. A. Hepatic superoxide dismutase (SOD) activity determined by assay kit for control (Ctrl), androgenized (Andro) or androgenized + E2 (Andro + E2) animals maintained on control Lieber-DeCarli (C-LDC) or ethanol-LDC (E-LDC) diet. B. Hepatic catalase activity determined by assay kit for Ctrl, Andro, Andro + E2 rats maintained on C-LDC or E-LDC diets. Activity levels were normalized to total protein. Data are presented as mean values ± SEM. *P<0.05.

Because generation of ROS is a key event in development and progression of ALD, alterations in antioxidant defenses were assessed by measuring superoxide dismutase (SOD) and catalase activities. SOD activity was unchanged in animals maintained on C-LDC diets. Conversely, in animals maintained on E-LDC diets, SOD activity was significantly decreased in the Andro group compared to Ctrl (Fig. 4A). Supplementation of E2 to Andro animals significantly increased SOD activity (Fig. 4A). No significant changes in catalase activity were observed among all groups (Fig. 4B).

### Androgenization Increased Fibrotic Gene Expression

**Figure 5:**
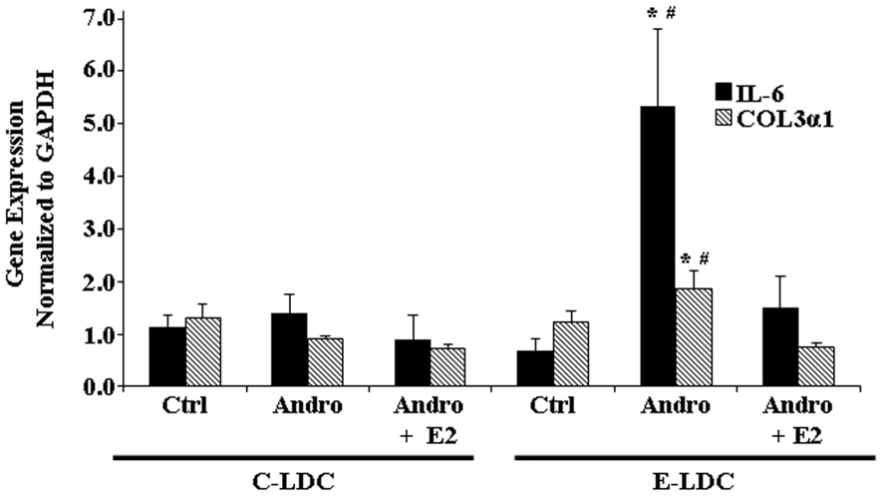
Effects of ethanol and androgenization on profibrotic gene expression. Quantitative Real-Time PCR analysis of Interleukin-6 (IL-6) (black bar) and collagen Type 3 (COL3α1) (striped bar) gene expression in control (Ctrl), androgenized (Andro) and Andro + estrogen (Andro + E2) animals maintained on control Lieber-DeCarli (C-LDC) or ethanol-LDC (E-LDC) diet. Gene expression was normalized to glyceraldehyde-3-phosphate dehydrogenase (GAPDH). Data are presented as mean values ± SEM. *P<0.05 *vs*. Ctrl + E-LDC; ^#^P<0.05 *vs*. Andro + E2 + E-LDC.

As ethanol consumption is a risk factor for developing hepatic fibrosis, which can be preceded by steatohepatitis, we next examined profibrotic gene expression by qRT-PCR. Differences in *IL-6* and *col3α1* gene expression were not observed in the three experimental groups maintained on C-LDC (Fig. 5). Up-regulation of *IL-6* and *col3α1* mRNA was observed in Andro animals on E-LDC compared to Ctrl on E-LDC (Fig. 5). Continuous exogenous E2 exposure in adult rats abrogated the androgenization effect of increased profibrotic gene expression (Fig. 5).

### Cytochrome P450 Expression is Modulated by Androgenization and Ethanol

**Figure 6:**
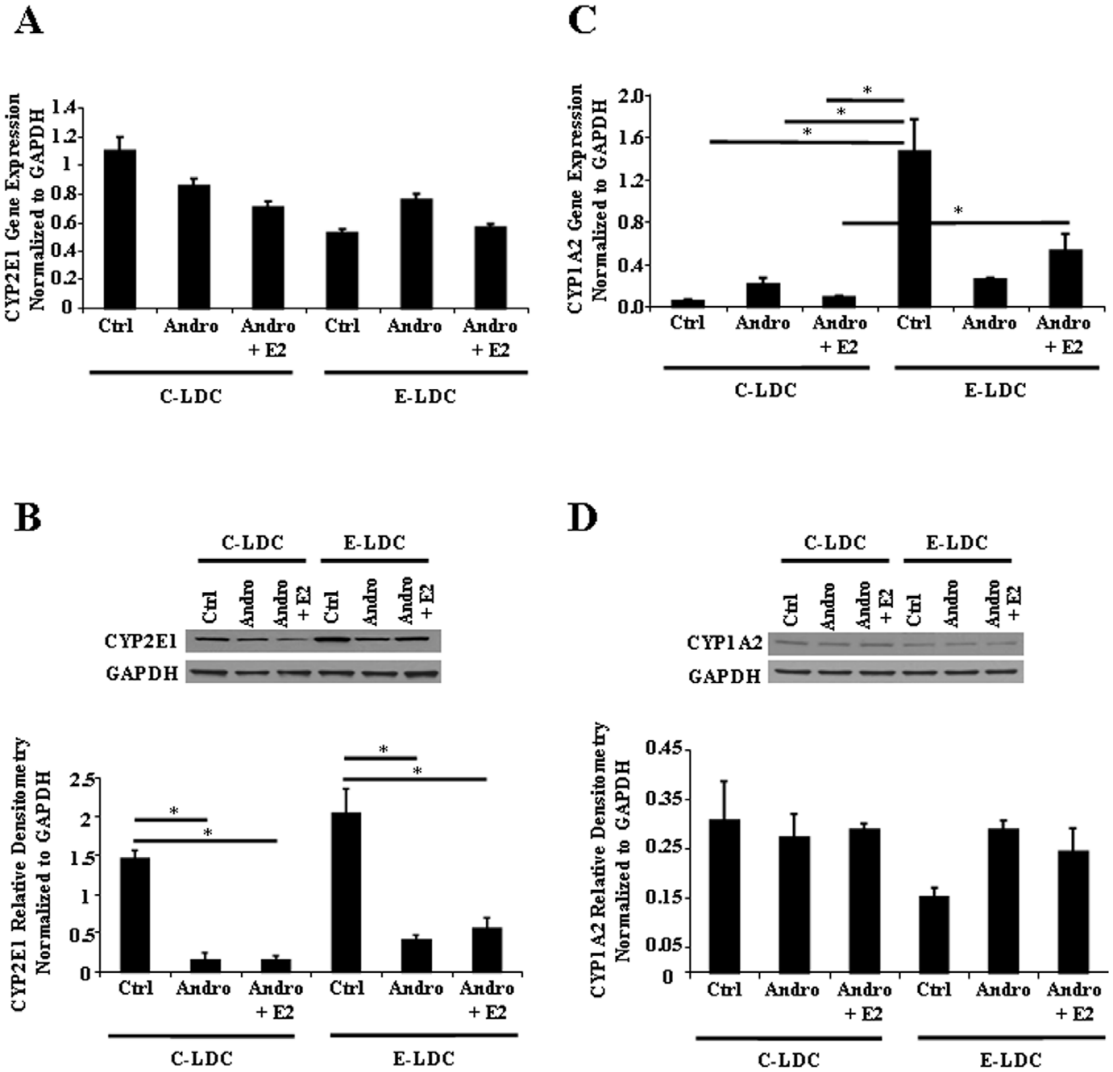
Effects of ethanol and androgenization on CYP450 expression. A. Quantitative Real-Time PCR analysis of CYP2E1 mRNA expression in control (Ctrl), androgenized (Andro) and Andro + estrogen (Andro + E2) rats maintained on control Lieber-DeCarli (C-LDC) or ethanol-LDC (E-LDC) diet. B. Representative CYP2E1 protein expression was determined by Western blot analysis (upper panel) and quantified by optical integrated volume (lower panel). Data are presented as mean values ± SEM. Standard square root transformation was performed prior to statistical analysis. *P<0.05. C. Quantitative Real-Time PCR analysis of CYP1A2 mRNA expression compared between groups described in A. Data are presented as mean values ± SEM. *P<0.05. D. Representative CYP1A2 protein expression was determined by Western blot analysis (upper panel) and quantified by optical integrated volume (lower panel). RNA and protein expression were normalized to GAPDH.

Chronic ethanol consumption induces hepatic microsomal CYP2E1 leading to increased generation of ROS [26]. Hormonal imprinting of liver enzymes is known to occur [27], which suggests that changes in sex specific expression of CYP450 family members may contribute to sex differences in ALD. Therefore, cytochrome P450 2E1 (CYP2E1) and cytochrome P450 1A2 (CYP1A2, a predominantly female expressed enzyme) protein expression were measured to determine effects of androgenization on ethanol metabolizing enzymes. *cyp2E1* expression was unchanged at the mRNA level across all experimental groups (Fig. 6A), but because CYP2E1 is predominantly regulated post-transcriptionally, protein expression was assessed. Androgenization significantly decreased CYP2E1 protein expression in animals maintained on the C-LDC diet, and a similar effect was observed in animals maintained on E-LDC (Fig. 6B). E2 implants did not significantly alter CYP2E1 expression in Andro groups maintained on either C-LDC or E-LDC diets (Fig. 6B). Significant up-regulation of *cyp1a2* mRNA with the addition of ethanol was observed in the Ctrl, but androgenization inhibited this effect (Fig. 6C). Additionally, E2 supplementation to Andro group increased *cyp1a2* expression in the ethanol treated animals compared to animals on C-LDC (Fig. 6C). In contrast to CYP2E1, no significant differences in CYP1A2 protein expression were detected between any of the experimental groups (Fig. 6D).

## Discussion

Our study demonstrated that inhibition of adult cyclic estrogen (E2) *via* neonatal androgenization exacerbated the deleterious effects of ethanol through increased hepatic steatosis (H&E and liver triglycerides). Additionally, androgenized animals on the ethanol Lieber-DeCarli diet (E-LDC) showed significant increases in lipid peroxidation (4-HNE and TBARS) compared to Ctrl animals on E-LDC, and decreased superoxide dismutase (SOD) activity in these animals further contributed to increased oxidative stress. Further, disruption of normal cyclic E2 up-regulated mRNA expression of profibrotic markers, *IL-6* and *col3α1*. Additionally, all indicators of liver injury were reversed by exogenous administration of E2, supporting a protective effect of continuous E2 exposure and/or supraphysiological dose of E2, observed only in animals on the ethanol diet, in the adult androgenized rat. Although androgenization exacerbated ethanol-induced liver injury (e.g. steatosis, lipid peroxidation, oxidative stress and profibrotic gene expression), only increased steatosis was observed in Ctrl animals on the E-LDC diet compared to control LDC (C-LDC), while all other liver injury markers were unchanged. This was unexpected since the four week LDC diet leads to mild liver injury (i.e. steatosis); however, a more severe form of liver injury is achievable by the intragastric feeding model [28] or a longer duration of ethanol feeding (i.e. eight weeks) which would result in increased liver damage as observed by increased inflammation, hepatic stellate cell activation and oxidative stress [19]. It will be of interest to determine if increased triglyceride accumulation by our model of androgenization with ethanol administration occurs in other tissues such as adipose to document if this is a general phenomenon or liver specific.

Since normal cyclic patterning of hormone release can program hepatic enzyme expression, CYP2E1 mRNA and protein were examined. Androgenization decreased CYP2E1 protein expression compared to Ctrl females on C-LDC and E-LDC diets, suggesting a relationship between cyclic E2 and expression of CYP2E1. Previous studies reported the deleterious effects of ethanol metabolism were independent of CYP2E1 expression, as CYP2E1 knockout mice were equally susceptible to ethanol-induced liver injury [13]. Our data also supports that CYP2E1 does not contribute to liver pathology since high E2 concentrations did not alter CYP2E1 expression between Andro and Andro + E2 groups, although pathology was changed. However, this does not preclude other sex-specific CYP450 enzymes in the progression of ALD, including CYP1A2, a predominantly female contributor to the microsomal ethanol oxidizing system [14], [16]. In our study, *cyp1A2* mRNA expression was up-regulated in Ctrl animals on E-LDC compared to C-LDC and androgenization abrogated this effect, while no significant difference in protein expression was detected among all groups. This discrepancy observed between protein and message may be due to the four week ethanol exposure. Longer duration of ethanol consumption may lead to protein expression levels similar to what was observed at the mRNA level. Additionally, since induction of the microsomal ethanol oxidizing system is commonly associated with chronic ethanol consumption, the four week duration of ethanol feeding as well as the ethanol administration model system used (e.g. Lieber-DeCarli diet) may account for lack of ethanol-induced expression of CYP2E1 in Ctrl animals on E-LDC compared to C-LDC. Ethanol-increased expression of CYP2E1 is largely regulated by a post-transcriptional mechanism due to protein stabilization [12]. Therefore, in addition to Western blot analyses, future studies should examine CYP2E1 and CYP1A2 enzyme activity. Collectively, these results support previous studies indicating CYP450 enzymes are not involved in ALD development or in the underlying sex differences [13]. Collectively, these findings suggest that cyclic E2 is not solely responsible for the observed sexual differences in ALD, but instead, variations in gonadal hormone regulation by the HPG axis may contribute to disease susceptibility.

Even though E2 has been implicated in development and progression of ALD, previous studies have reported protective effects of E2 associated with increased antioxidant activities and subsequent reduction in lipid peroxidation in non-alcoholic liver diseases including hepatitis C virus, hepatocellular carcinoma and sepsis [29], [30], [31]. Similarly, improvement of hepatic steatosis by E2 has been observed in an animal model of obesity and alcohol [32]. Specifically, high circulating levels of E2 showed suppressive effects in a fibrotic animal model [33]. As E2 is a strong endogenous antioxidant [34], the mechanism of action has been attributed to reduced levels of ROS generation, lipid peroxidation, and activation of activator protein (AP)-1 and NFκB [34], [35], [36]. Interestingly, in our study continuous exogenous administration of E2 to adult androgenized female rats on the alcohol diet, which increased E2 concentration by 7-fold compared to animals on control diet (571.16±52.3 vs 81.17±12.1), displayed protective effects as marked by decreased steatosis, lipid peroxidation and profibrotic gene expression. Noteworthy, is the high concentration of plasma E2 in Andro + E2 animals on the alcohol diet compared to Andro + E2 on the control diet. Chronic alcohol consumption alters hormone levels, and our data suggests that alcohol may impede metabolism of E2 in the liver leading to increased plasma levels. Further, SOD activity was increased in androgenized animals implanted with E2, showing protective effects of this hormone. Even though elevated SOD activity leads to increased generation of H_2_O_2_, all groups displayed similar catalase activity; therefore, differences in H_2_O_2_ cellular concentrations should not contribute to overall oxidative stress. Although exogenous E2 decreased catalase activity in Andro animals on C-LDC compared to all other groups, this change did not reach significance. Additionally, exogenous E2 had no effect on CYP2E1 expression. Therefore, these data suggest that timing and pattern of E2 release regulated by the HPG axis plays a critical role in development and progression of ALD in adulthood.

Androgenization is a well-established method of hypothalamic-pituitary masculinization and inhibition of cyclic E2 [constant baseline levels of E2 are observed (Table 1)] [23], [24], [25]; therefore, we chose this model to elucidate the role of cyclic E2 in ALD progression. Since previous studies implicating E2 in ALD development regulated E2 expression through OVX and antiestrogen administration (selective E2 receptor modulators), and did not observe complete reversal of the damaging effects of ethanol, the role of E2 remains to be fully elucidated [7], [8]. In OVX ovarian tissue is completely removed, and the contribution of other ovarian factors, such as progesterone and inhibin, cannot be discounted. Androgenization inhibits adult cyclic E2 production in the presence of intact ovaries. However, androgenization not only inhibits cyclic E2, but also disrupts the HPG axis, therefore, implicating a role for other sex hormones (e.g. LH and FSH) in ALD. Studies have speculated differences in androgenization and OVX models with respect to LH concentrations. In androgenized rats E2 feedback on the hypothalamus may prevent pituitary LH storage, while in OVX animals increased amounts of LH and FSH may occur due to lack of hormonal feedback resulting in release of these gonadotropins [18]. Chronic ethanol consumption alters serum LH and FSH, confirming an ethanol effect on pituitary hormone release [37], [38], [39]. Therefore, it will be crucial to determine the contribution of additional sex steroid hormones regulated by the HPG axis on the development of ALD. Further, it is possible that postnatal androgenization may also lead to other endocrine changes in addition to E2 cyclicity in adults or higher than normal prepubertal estrogen production leading to such problems as metabolic dysfunction and insulin resistance that would impact liver physiology.

Many factors, including environmental endocrine disruptors and toxins, can disrupt the HPG axis. Postnatal exposure to bisphenol A (BPA; an environmental estrogenic compound) has been shown to induce anovulation and infertility in female rats [40], [41]. In adulthood, ethanol can directly modulate sex steroid hormones through aromatization of testosterone to E2, resulting in feminization of male alcoholics [42], [43]. The HPG axis is not only altered by environmental factors and toxins, but in clinical disorders such as polycystic ovary syndrome (PCOS), which affects 6-8% of premenopausal women [44]. PCOS leads to hypersecretion of androgens and altered patterning of sex steroid hormone release [45]. Interestingly, the incidence of non-alcoholic fatty liver disease in women with PCOS is increased, further supporting the role of the HPG axis in susceptibility to fatty liver disease [46]. All aforementioned challenges to the HPG axis postnatally could alter adult liver physiology of both males and females, potentially impacting development and progression of ALD.

In summary, cyclic E2 and possibly other sex steroid hormones regulated by the HPG axis may contribute to the development and progression of ALD, conferring sex differences. Moreover, timing and pattern of E2 delivery can contribute to severity of ALD. Understanding the interplay of sex steroid hormone exposure and the HPG axis in liver physiology is critical to predicting susceptibility to ALD and possibly other hepatotoxins.
